# Genome Editing of Golden SNP-Carrying Lycopene Epsilon-Cyclase (*LcyE*) Gene Using the CRSPR-Cas9/HDR and Geminiviral Replicon System in Rice

**DOI:** 10.3390/ijms231810383

**Published:** 2022-09-08

**Authors:** Jong Hee Kim, Jihyeon Yu, Hee Kyoung Kim, Jin Young Kim, Me-Sun Kim, Yong-Gu Cho, Sangsu Bae, Kwon Kyoo Kang, Yu Jin Jung

**Affiliations:** 1 Division of Horticultural Biotechnology, School of Biotechnology, Hankyong National University, Anseong 17579, Korea; 2 Division of Life Sciences, Korea Polar Research Institute, Incheon 21990, Korea; 3 Department of Crop Science, Chungbuk National University, Cheongju 28644, Korea; 4 Department of Biomedical Sciences, Seoul National University College of Medicine, Seoul 03080, Korea; 5 Institute of Genetic Engineering, Hankyong National University, Anseong 17579, Korea

**Keywords:** gene replacement, homology-directed repair (HDR), CRISPR-Cas9, geminiviral replicons, genome editing, *LcyE*, rice

## Abstract

Lycopene epsilon-cyclase (*LcyE*) is a key enzyme in the carotenoid biosynthetic pathway of higher plants. Using the CRSPR/Cas9 and the geminiviral replicon, we optimized a method for targeted mutagenesis and golden SNP replacement of the *LcyE* gene in rice. We have exploited the geminiviral replicon amplification as a means to provide a large amount of donor template for the repair of a CRISPR-Cas-induced DNA double-strand break (DSB) in the target gene via homology-directed repair (HDR). Mutagenesis experiments performed on the Donggin variety achieved precise modification of the *LcyE* loci with an efficiency of up to 90%. In HDR experiments, our target was the *LcyE* allele (LcyE-H523L) derived from anther culture containing a golden SNP replacement. The phenotype of the homologous recombination (HR) mutant obtained through the geminiviral replicon-based template delivery system was tangerine color, and the frequency was 1.32% of the transformed calli. In addition, the total carotenoid content of the *LcyE*sg2-HDR1 and *LcyE*sg2-HDR2 lines was 6.8–9.6 times higher than that of the wild-type (WT) calli, respectively. The reactive oxygen species content was lower in the *LcyE*sg2-HDR1 and *LcyE*sg2-HDR2 lines. These results indicate that efficient HDR can be achieved in the golden SNP replacement using a single and modular configuration applicable to different rice targets and other crops. This work demonstrates the potential to replace all genes with elite alleles within one generation and greatly expands our ability to improve agriculturally important traits.

## 1. Introduction

Targeted genome editing has become a powerful genetic tool for studying gene function and for introducing new functions or modifying the genome to correct defective genes. Therefore, it is becoming a very important tool in plant breeding compared to the random mutagenesis and recombination techniques that have been used previously to generate novel genetic variation. Accurate genome editing has been achieved through the use of targeted double-strand break (DSB) induction through the clustered regularly spaced short palindromic repeats (CRISPR) and CRISPR-associated protein 9 (Cas9) systems [1]. To date, many studies have demonstrated the potential of CRISPR-Cas9-mediated genome editing to induce specific indel mutations, gene targeting (GT), and controlled gene expression [2,3,4,5,6,7,8,9,10]. However, GT or gene replacement is possible but still not a routine method due to its low frequency. Various methods have been reported over the years to increase the frequency of HDR between genomic targets and extrachromosomal homologous donors [11,12,13,14]. In plants, HDR or gene replacement required the coordination between the induction of DSBs from genomic targets and the existence of donor recovery templates in the cells. However, in the plant genome engineering field, the non-homologous end joining DNA repair pathway (NHEJ) mediated by the knockout method is still favored over the HDR method. The reason for this is that it is difficult to deliver a sufficient copy number of donor repair templates (DRT) in the cell for HDR, and the DRT stability in the cell is not guaranteed. It is quite challenging to deliver a DRT into a plant for the HDR-mediated repair of a DSB. Recently, Wang et al. [15] reported that they established a geminiviral replicon-based donor template delivery system to increase the frequency of gene knock-in in rice. Due to the high copy number and single-stranded DNA nature of the geminiviral genome, these genomes can be used as DNA template carriers for GT in plants. To date, successful gene targeting with the geminiviral replicon system required selection markers associated with the edited allele, indicating that targeting plant genes without the use of selection markers is still a challenge [16,17,18]. Therefore, GTs in plants, especially alleles without selectable markers, still need to be improved with many studies. Carotenoids are synthesized in plastids and accumulated as red, orange, and yellow pigments, among which β-carotene, lycopene, and lutein are industrially important components as powerful antioxidants [19]. The cyclization of lycopene ends catalyzed by lycopene *β*-cyclase and lycopene *ε*-cyclase forms *β*-carotene or *α*-carotene. Wakasa et al. [20] reported the characteristics of two callus lines showing orange and tangerine colors by repeatedly subculturing anther culture-derived mutant calli. Ishihara et al. [21] showed that the tangerine callus line is the *LcyE* gene (H523L), which carries a single nucleotide polymorphism responsible for His to Leu substitution at amino acid position 523, exhibiting a dramatically higher carotenoid content than WT calli. Here, we created HDR callus mutants (*LcyE*sg2-HDR1 and *LcyE*sg2-HDR2) with golden SNP-carrying *OsLcyE* genes using the CRSPR/Cas9 and the geminiviral replicon system. Our results showed that the *LcyE*sg2-HDR1 and *LcyE*sg2-HDR2 lines increased the content of total carotenoids and reduced ROS accumulation under salt stress conditions.

## 2. Results

### 2.1. Efficient Allelic Replacement by the Geminiviral Replicon for Targeted Modifications

**Figure 1:**
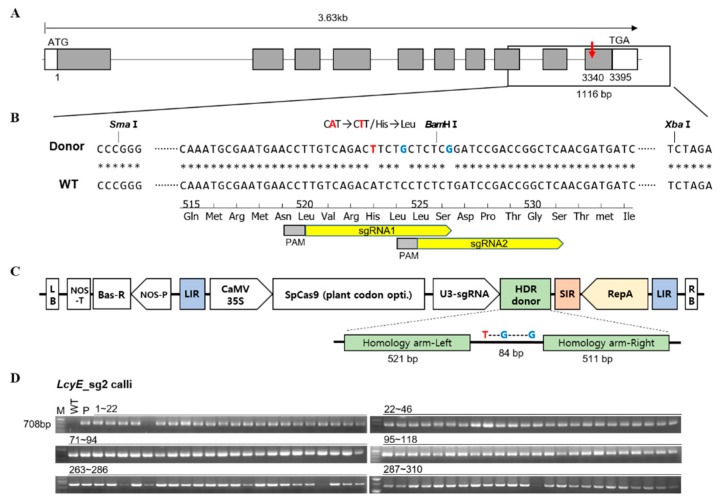
(**A**) Schematic representation of the *LcyE* gene and targeting sites. (**B**) The nucleotides highlighted in red correspond to intended modifications for SNP, and in blue are the silent mutations to avoid re-cutting and to generate cleaved amplified polymorphic sequence (CAPS) markers. Asterisks indicate identical nucleotides. The cutting sites of the two guide RNAs used in this study are indicated by two yellow arrows with PAM. (**C**) Schematic representation of the CRISPR/Cas9 vector and the homologous DNA donor template construction. (**D**) PCR analysis of the bar gene and the NOS terminator region (primer set NOS-bar F/R) in the rice callus introduced with the pGemBos::*LcyE* vector. M: 1 kb DNA ladder; WT: wild type; P: pGemBos::*LcyE* plasmid vector.

**Figure 2:**
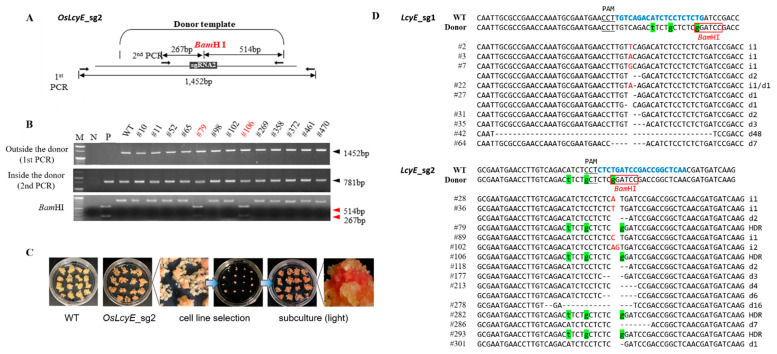
(**A**) Position of key SNP replacement of the *LcyE* targeted site (*Bam*HI restriction enzyme site was created inside the donor). (**B**) Screening by using PCR and restriction enzyme analysis. The second PCR products were digested with the *Bam*HI restriction enzyme. M: 1 kb DNA ladder; N: negative control; P: pGemBos::*LcyE*_sg2 plasmid vector; WT: wild type. (**C**) Phenotype of edited callus lines using the CRISPR-Cas9-based geminiviral replicon system in rice. (**D**) Mutation patterns of the target sequence region through deep sequencing. The target DNA sequence of *LcyE*_sg1 and *LcyE*_sg2 is shown in the WT with blue text at the top of the aligned sequences. The PAM sequences are underlined. Deletions are indicated as dashes; insertions are in red; and substitutes are in green highlighter. Indel sizes are shown on the right (i, insertion; d, deletion).

To accurately replace an existing allele in the genome with an elite allele without a selection marker, we improved the HDR strategy in the following ways using the geminiviral replicon previously reported by Dahan-Meir et al. [22]. First, we mutated the protospacer adjacent motif (PAM) site in the core sequence of the DRT to prevent the Cas9/gRNA from cleaving the DRT when the HDR was successful. We also created a recognition site of *BamH*I in the DRT as a marker to identify HDR events (Figure 1A,B and Supplementary Figure S1). According to our strategy, we constructed a Ti-plasmid vector that expresses both Cas9 and gRNAs, which generate DSBs at the targeted genomic sites and release a DRT from the plasmid (Figure 1C, Supplementary Table S1, and Figure S2). Therefore, these T-plasmid vectors contain all the components for replacement with *OsLcyE* (H523L) (Figure 1). The transformed calli were subcultured three times in 2N6 medium containing 6 mg/L of phosphinothricin. First, in order to determine whether the T-DNA region was introduced, the expected band was amplified in 95% of the results of the PCR analysis using the primer set NOS-barF/R (Figure 1D and Supplementary Figure S3). To confirm HDR events, a total of 387 transgenic calli were digested with *Bam*HI or subjected to deep sequencing. Notably, HDR occurred only in *LcyE*sg2; 1.32% (4/304) of the transgenic calli showed the intended precise HDR events (Figure 2D, Table 1). In addition, the majority (54.5%) of the mutation types were generated through NHEJ (Figure 2D and Table 1). Therefore, two of these four calli were selected and named *LcyE*sg2-HDR1 and *LcyE*sg2-HDR2, respectively, and were subcultured to analyze their characteristics (Figure 2A–C).

### 2.2. Protein Model According to the Golden SNP Replacement of OsLcyE

**Figure 3:**
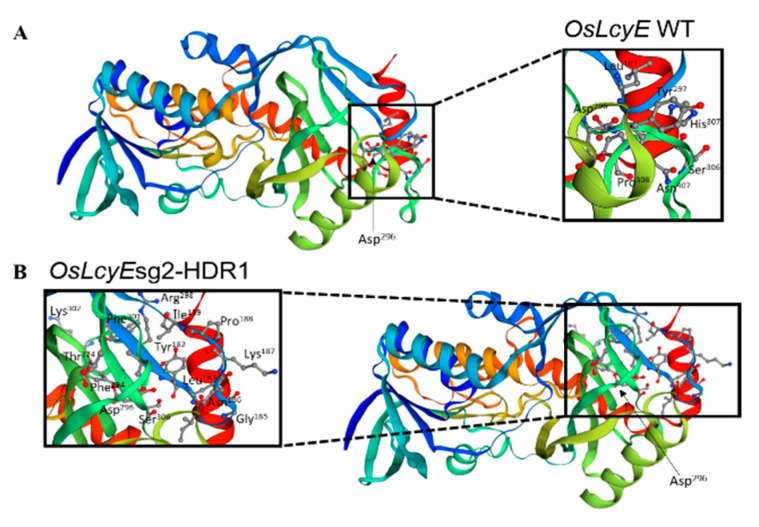
Structural comparison of the wild-type *OsLcyE* and the HDR-mutated version *OsLcyE*. (**A**) Wild-type *OsLcyE* showing the position of Asp^296^. The black box shows all of the key amino acids (Leu^183^, Asp^296^, Tyr^297^, Ser^306^, His^307^, Pro^308^, and Asn^407^). (**B**) Mutant *OsLcyE* showing the position of Asp^296^. The black box shows all of the key amino acids (Tyr^182^, Leu^183^, Gly^185^, Asn^186^, Lys^187^, Pro^188^, Ile^189^, Phe^294^, Asp^296^, Arg^298^, Phe^301^, Lys^302^, Ser^306^, and Thr^324^).

In the *OsLcyE* gene, the golden SNP is replaced by HDR in exon 8 using the CRISPR-Cas9 system, resulting in an amino acid change from His to Leu. The *LcyE*sg2-HDR1 and *LcyE*sg2-HDR2 lines were investigated to find changes at the protein level that might explain the change of LcyE activity. By translating the mutant sequences and aligning them with the wild-type LcyE, we were able to identify key functional residues. We then used homology modeling to determine the effect of mutations on the structure and key functional residues of the enzyme (Figure 3). The *LcyE*sg2-HDR1 and *LcyE*sg2-HDR2 lines were changed from H to L at 523. Comparative models of the wild-type LcyE and mutant versions showed that the frame shift was caused by the replacement of the catalytic triad and the His^523^ residue, which plays an important role in stabilizing the catalytic center (Figure 3). The LcyE structure of the wild type is linked by peptide chains of Leu^183^, Asp^296^, Tyr^297^, Ser^306^, His^307^, Pro^308^, and Asn^407^, whereas the LcyE structure of the HDR mutant lines is composed of Tyr^182^, Leu^183^, Gly^185^, Asn^186^, Lys^187^, Pro^188^, Ile^189^, Phe^294^, Asp^296^, Arg^298^, Phe^301^, Lys^302^, Ser^306^, and Thr^324^, which were converted into peptide chains (Figure 3). These mutant lines were replaced with golden SNP in the epsilon Lyc motif domain, resulting in slight changes in the enzymatic structure (Figure 3B). Thus, changes in the LcyE activity in the *LcyE*sg2-HDR1 and *LcyE*sg2-HDR2 lines reflect the de novo acquisition of a regulatory C-terminal domain that determines the substrate specificity of the catalytic center.

### 2.3. Carotenoid Analysis in the LcyEsg2-HDR1 and LcyEsg2-HDR2 Lines

The accumulated amounts of lycopene and *β*-carotene were determined by HPLC analysis using a standard curve obtained from an authentic compound. Unidentified carotenoid content eluted at 10.2, 30.2, 33.1, and 35.3 min was determined based on a standard curve for *β*-carotene. The *LcyE*sg2-HDR1 and *LcyE*sg2-HDR2 lines accumulated mainly lycopene with smaller amounts of carotene, such as lutein and α-carotene. However, the WT line accumulated mainly *β*-carotene with another carotene (Table 2). The total amount of carotenoids in the *LcyE*sg2-HDR1 and *LcyE*sg2-HDR2 lines was approximately 15 times larger than that in WT calli.

### 2.4. Reduced ROS Accumulation under Salt Stress Conditions

**Figure 4:**
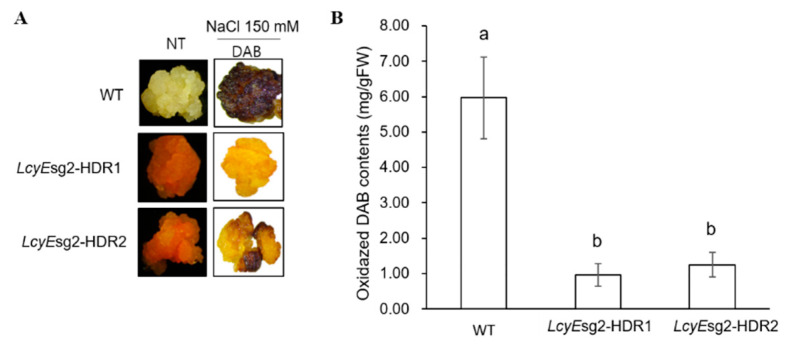
Salt-induced oxidative stress in the WT, *LcyE*sg2-HDR1, and *LcyE*sg2-HDR2 lines was visualized by DAB staining in rice. (**A**) Visible damage in the WT, *LcyE*sg2-HDR1, and *LcyE*sg2-HDR2 lines after stress treatment with 150 mM NaCl by DAB staining. NT: picture of the calli of each line before salt treatment. (**B**) Oxidized DAB contents in the WT, *LcyE*sg2-HDR1, and *LcyE*sg2-HDR2 lines incubated at 150 mM NaCl. Bars indicate the standard error of the mean.

Given the high total carotenoid accumulation, we examined the effects of the *LcyE*sg2-HDR1 and *LcyE*sg2-HDR2 lines on the tolerance to oxidative stress induced by salt. Salt-induced oxidative stress in the calli was visualized by DAB staining, which turns to a dark brown when oxidized by H_2_O_2_. The *LcyE*sg2-HDR1 and *LcyE*sg2-HDR2 lines maintained a tangerine color, whereas the WT calli exhibited a dark brown color (Figure 4A). These results show that oxidative species and DAB reactants were reduced in response to NaCl stress in the *LcyE*sg2-HDR1 and *LcyE*sg2-HDR2 lines (Figure 4B).

## 3. Discussion

To date, targeted mutations using the CRISPR-Cas9 system have already been shown in several plant species, including rice [23,24,25]. Among them, non-homologous end joining (NHEJ), which does not require a homologous template, is a pathway to repair double-strand breaks in DNA. However, since homology-directed repair requires homologous sequences to guide the repair, the substitution frequency is very low, so it is not yet routinely used. Therefore, many researchers have tried to increase the frequency of gene replacement using HDR [11,12,13,14,26]. For HDR to be possible in plants, NHEJs that induce DSBs in the target genes must first be readily available. Second, so that the donor repair template (DRT) can be delivered very easily, the residence time of the intracellular donor, the donor size, and the culture conditions must be adjusted. Various strategies have been used to increase the availability of DRT to precisely modify plant genomes [15,17,27]. Despite such efforts, we believe that the reason HDR is not used routinely is because it is difficult to deliver a sufficient copy number of intracellular donor repair templates (DRT) for HDR, and intracellular DRT stability is not guaranteed. In this study, we achieved a homologous HDR frequency of 1.32% in rice calli using the CRSPR/Cas9 and geminiviral replicon systems (Table 1 and Figure 1 and Figure 2D). Our HDR strategy is a system that allows for primary phenotypic selection by replacing the golden SNP (H523L) in *OsLcyE*, allowing for immediate color change of the calli (Figure 2A–C). In addition, when HDR occurs, base adjustments were made to the donor DNA so that it could be distinguished by the restriction enzyme *Bam*HI (Figure 1B and Figure 2A,B). The gemini viruses used in this experiment are plant viruses with a single-stranded circular DNA genome (2.5–3.0 kb) and a broad host range, including monocot and dicot plants. Once infected, they are known to generate numerous replicons via rolling circle replication that can serve as repair templates during HDR [27,28]. To date, the frequency of successful HDR without antibiotic selection markers in donor DNA has been reported to be 0.07% in tomatoes [29] and 0.2–0.8% in rice [14,30,31]. In addition, the HDR success rate was very low in experiments using the herbicide tolerance gene for DRT in *Arabidopsis* [32]. Therefore, it is judged that the HDR system without antibiotics in plants is difficult to utilize for plant breeding unless the frequency of HDR is increased. In this respect, this study differs from previous reports in that there are no selectable markers in HDR, and it has shown promise to increase the success rate of HDR. Therefore, aspects of this study can be used for research to improve the frequency of HDR, such as the copy number of donors and the intracellular residence time for HDR. In particular, the *LcyE*sg2-HDR1 and *LcyE*sg2-HDR2 lines accumulated mainly lycopene with smaller amounts of carotene, such as lutein and α-carotene (Table 2). The cause is thought to be due to differences in carotenoid biosynthesis activity or binding activity to the target protein according to the replacement of golden SNP in *OsLcyE*. According to our previous report, changes in carotenoid levels increased the salt stress tolerance [33]. Therefore, we investigated the effect of the *LcyE*sg2-HDR1 and *LcyE*sg2-HDR2 lines, which reduced ROS accumulation under salt stress conditions (Figure 4). Taken together, it is thought that the carotenoid biosynthesis intermediate obtained from the *LcyE*sg2-HDR1 and *LcyE*sg2-HDR2 lines produced higher amounts of antioxidants than the WT through cooperation with reactive oxygen species that reduced cell damage under salt stress. Although the HDR frequency is still much lower than NHEJ, the four independent precise HDR lines obtained are likely sufficient for the purpose of crop improvement. Therefore, our HDR system has broad applications in plant breeding, such as replacing disease susceptibility with disease resistance alleles in the same species that does not require back crossing or modifying key functional genes. In addition, this work demonstrates that the use of CRISPR/Cas9-mediated HDR for target allele replacement in rice without associative drag is feasible, effective, and significantly accelerates field-ready trait introgression in crop improvement.

## 4. Materials and Methods

### 4.1. Construction of Gene-Editing Vectors and Agrobacterium-Mediated Transformation

pGemBos, a plant binary vector for CRISPR-induced HDR, was modified from pTC217 (pTC217 was a gift from Daniel Voytas, Addgene: #70018) [26]. An AtU6 promoter and a GT donor were removed by a Cas9-RNP cut in vitro, and an sgRNA cassette drive by an OsU3 promoter and a multi-cloning site (MCS) for donor insertion were introduced using the Gibson assembly. A Basta resistance gene, phosphinothricin acetyltransferase, controlled by an NOS promoter and a terminator, was also introduced by an *Mlu*I cut and the Gibson assembly for the antibiotic selection in plants. Two CRISRP-Cas9 target sites in the *OsLcyE* sequences were selected using the Cas-designer (http://www.rgenome.net/cas-designer/ accessed on 3 February 2020) [34] (Supplementary Figure S2). For CRISPR target sequences regarding cloning, annealed pairs of oligonucleotides, synthesized by Bioneer Co., Ltd. (Dajeon, Korea), were cloned to pGemBos by a *Bsa*I cut. HDR donor sequences with golden SNP replacement mutations, and silence mutations at the target site were synthesized (Bioneer, Dajeon, Korea) and introduced to MCS by a *Sma*I cut. The constructs thus obtained were transformed into a rice embryogenic callus using *Agrobacterium tumefaciens* strain EHA105, as previously described [34]. The transformed callus was selected using 6 mg/L of phosphinothricin and confirmed by PCR analysis, as previously reported [6,7]. To verify the target site mutation, PCR amplicons were subjected to MiniSeq paired-end read sequencing (Illumina, San Diego, CA, USA) and analyzed using the Cas-Analyzer (https://www.rgenome.net/cas-analyzer accessed on 4 September 2020) [35]. All transgenic callus lines performed several subcultures and were maintained in 2N6 medium [6], described previously (Figure 2C).

### 4.2. Total DNA Extraction and Mutation Detection

Total DNA extraction was performed by crushing 0.5 g of callus under liquid nitrogen with an electric drill in a 1.5 mL Eppendorf tube. A volume of 700 μL of extraction buffer (0.8 M NaCl; 0.15 M sorbitol; 0.12 M Tris-HCl, pH 7.5; 22 mM EDTA; 0.8% CTAB; and 0.8% sodium lauroyl sarcosinate) was added to each tube and incubated for 20 min at 65 °C. A volume of 800 μL of chloroform-isoamyl alcohol (24:1) was added to each tube and was centrifuged at 13,000 rpm for 5 min. This step was repeated twice. A volume of 1.4 mL of storage buffer (80% EtOH and 0.2 M sodium acetate) was added to the supernatant in a new 2 mL Eppendorf tube. DNA precipitation was performed by centrifugation at 13,000 rpm for 20 min at 4 °C. The pellet was re-suspended in 50 uL of H_2_O. The extracted genomic DNA was then used as a template to amplify the relevant fragments from each of the target genes by using primers (Supplementary Table S2) flanking the target sites. The standard PCR conditions were as follows: 94 °C for 3 min; 94 °C for 30 s; 56 °C for 30 s; 72 °C for 30 s for 35 cycles; and 72 °C for 10 min. The PCR products were directly sequenced by NGS technology using internal sequencing primers (Supplementary Table S2) to identify mutations. The mutation rate of each target is calculated as a ratio, which is the number of transgenic calli edited in each target to the total number of transgenic calli obtained.

### 4.3. Phenotypic Observations of the Golden SNP Replacement LcyE Mutant Calli

The phenotype of the HDR mutant calli substituted with H523L in the *OsLcyE* gene was confirmed by light microscopy in the second subculture after transformation and was photographed.

### 4.4. Homology Modeling and Protein Structure Analysis

The *LcyE* sequence was translated, and the mature *OsLcyE* polypeptide (523 amino acids) was aligned with the mutant version from the *LcyE*sg2-HDR1 and *LcyE*sg2-HDR2 lines using the Unipro UGENE alignment platforms Kalign, MUSCLE, and ClustalW. Homology modeling was performed using Phyre2. The model of the mutant protein was overlaid on the wild-type version using DS Visualizer.

### 4.5. Extraction of Pigments and Measurement of Absorption Spectra

After 28 days of subculture, the callus (0.1 g) was homogenized by crushing 0.5 g of the callus under liquid nitrogen. Acetone (1 mL) was added to the homogenate and mixed well. The supernatant after centrifugation at 13,000×*g* rpm for 5 min at 4 °C was collected. This procedure was repeated, and the second supernatant was combined with the first supernatant, dried, and dissolved in 500 μL acetone. This solution was diluted with acetone, and the absorption spectrum was measured using a spectrophotometer Ultrospec 3000 (Pharmacia Biotech, Seoul, Korea).

### 4.6. Analysis of Carotenoid Contents

Carotenoids were extracted from callus tissues using a 0.01% solution of butylated hydroxytoluene in acetone and analyzed using an Agilent 1100 HPLC system (Hewlette Packard, Palo Alto, CA, USA) according to the method described by ref. [36].

### 4.7. H_2_O_2_ Assays

To measure cellular levels of H_2_O_2_, each callus was incubated in a 1 mg mL^−1^ solution of 3,3-diaminobenzidine (DAB)-HCl (pH 3.8) for 5 h at 25 °C under light conditions according to the methods of [37]. For H_2_O_2_ assays, the DAB solution was measured as the absorbance at 460 nm. Oxidized DAB concentrations were calculated from the standard curve of DAB.

### 4.8. Statistical Analysis

All experiments included at least three independent technical and biological replicates, and data are presented as means ± standard error of the mean (SEM). All data analyses and processing were performed using Microsoft Excel 2019 (Microsoft Corporation, Anseong, Korea) and RStudio. Data for ROS experiments under salt stress conditions for the WT, *LcyE*sg2-HDR1, and *LcyE*sg2-HDR2 lines were compared using one-way ANOVA and Tukey’s HSD. An analysis of variance was performed to look at the variation between groups and obtained the least significant difference at *p* < 0.05.

## Figures and Tables

**Table 1:** HDR efficiency and mutation types for the *OsLcyE* gene using the CRISPR-Cas9-based geminiviral replicon system.

Targeted Region	No. of Examined Calli	DNA Repair Pathway	No. of Edited Calli (%)	Mutant Type	No. of Mutation
*LcyE*-sg1	83	NHEJ	48 (57.8%)	Homo	8
				Bi-allelic	13
				Hetero	27
		HDR	0 (0%)	-	-
*LcyE*-sg2	304	NHEJ	169 (54.5%)	Homo	24
				Bi-allelic	46
				Hetero	93
		HDR	4 (1.32%)	Homo	4

**Table 2:** The content of carotenoids in *LcyE*sg2-HDR1, *LcyE*sg2-HDR2 mutant, and WT calli.

Lines	Lycopene		Lutein		13Z-*β*-Carotene		α-Carotene		*β*-Carotene		9Z-*β*-Carotene		Others		Total	
	Mean	SD	Mean	SD	Mean	SD	Mean	SD	Mean	SD	Mean	SD	Mean	SD	Mean	SD
WT	1.71	0.22	2.31	0.71	0.29	0.04	0.67	0.13	0.38	0.18	0.42	0.08	9.33	1.43	15.11	2.79
*LcyE*sg2-HDR1	205.1	36.0	2.87	0.10	0.23	0.01	1.08	0.41	0.09	0.01	0.44	0.02	21.11	1.21	230.92	37.76
*LcyE*sg2-HDR2	217.2	41.2	2.93	0.81	0.31	0.01	1.34	0.81	0.14	0.01	0.39	0.01	24.01	1.80	246.32	44.65

Data are expressed as means (the average value of content for dry weight) and SDs (the standard deviation value) of three independent experiments.

## Data Availability

Not applicable.

## Supplementary Materials

The following supporting information can be downloaded at: https://www.mdpi.com/article/10.3390/ijms231810383/s1.
