# Supplementary material for: Genome Editing of Golden SNP-Carrying Lycopene Epsilon-Cyclase (LcyE) Gene Using the CRSPR-Cas9/HDR and Geminiviral Replicon System in Rice
Source: Int J Mol Sci. 2022 Sep 8;23(18):10383. doi: 10.3390/ijms231810383 (PMC9499184; doi:10.3390/ijms231810383)
Supplement: Supplementary file 1 [file ijms-23-10383-s001.zip › Supplementary Figures and tables.pdf]

Supplementary Data

Genome Editing of Golden SNP-Carrying Lycopene Epsilon-Cyclase (*LcyE*)/HDR Gene using the CRSPR-Cas9 and Geminiviral Replicon System in Rice

Jong Hee Kim, Jihyeon Yu, Hee Kyoung Kim, Jin Young Kim, Me-Sun Kim, Yong-Gu Cho, Sangsu Bae, Kwon Kyoo Kang and Yu Jin Jung *

Contents

Table S1. Design of sgRNAs for HDR by CRISPR-Cas9 system in rice. using the Cas-designer (http://www.rgenome.net/cas-designer/). Under line is PAM sequence.

Figure S1. Design of donor template using the CRSPR-Cas9 and geminiviral replicon system.

Figure S2. Ti-plasmid vector construction using the CRSPR-Cas9 and geminiviral replicon system (A) pGemBos vector construction (B) Confirmation of donor template into pGemBos::*LcyE* vector (C) Confirmation of sgRNA by sequencing analysis.

Figure S3. Confirmation of T-DNA insertion from calli obtained through HDR experiment in rice. M: 1kb DNA ladder; WT: wild-type; P: pGemBos::*LcyE* plasmid vector.

Table S2. The primers list used in this study.

Supplementary Table S1. Design of sgRNAs for HDR by CRISPR-Cas9 system in rice. using the Cas-designer (http://www.rgenome.net/cas-designer/). Under line is PAM sequence.

sgRNA	RGEN Target (5' to 3')	Direction	GC content (%, w/o PAM)	Out-of frame score	Mismatches			
					0	1	2	3
sgRNA1	CAGAGAGGAGATGTCTGACA<u>AGG</u>	-	50.0	67.8	1	0	0	0
sgRNA2	TTGAGCCGGTCGGATCAGAG<u>AGG</u>	-	60.0	77.9	1	0	0	0

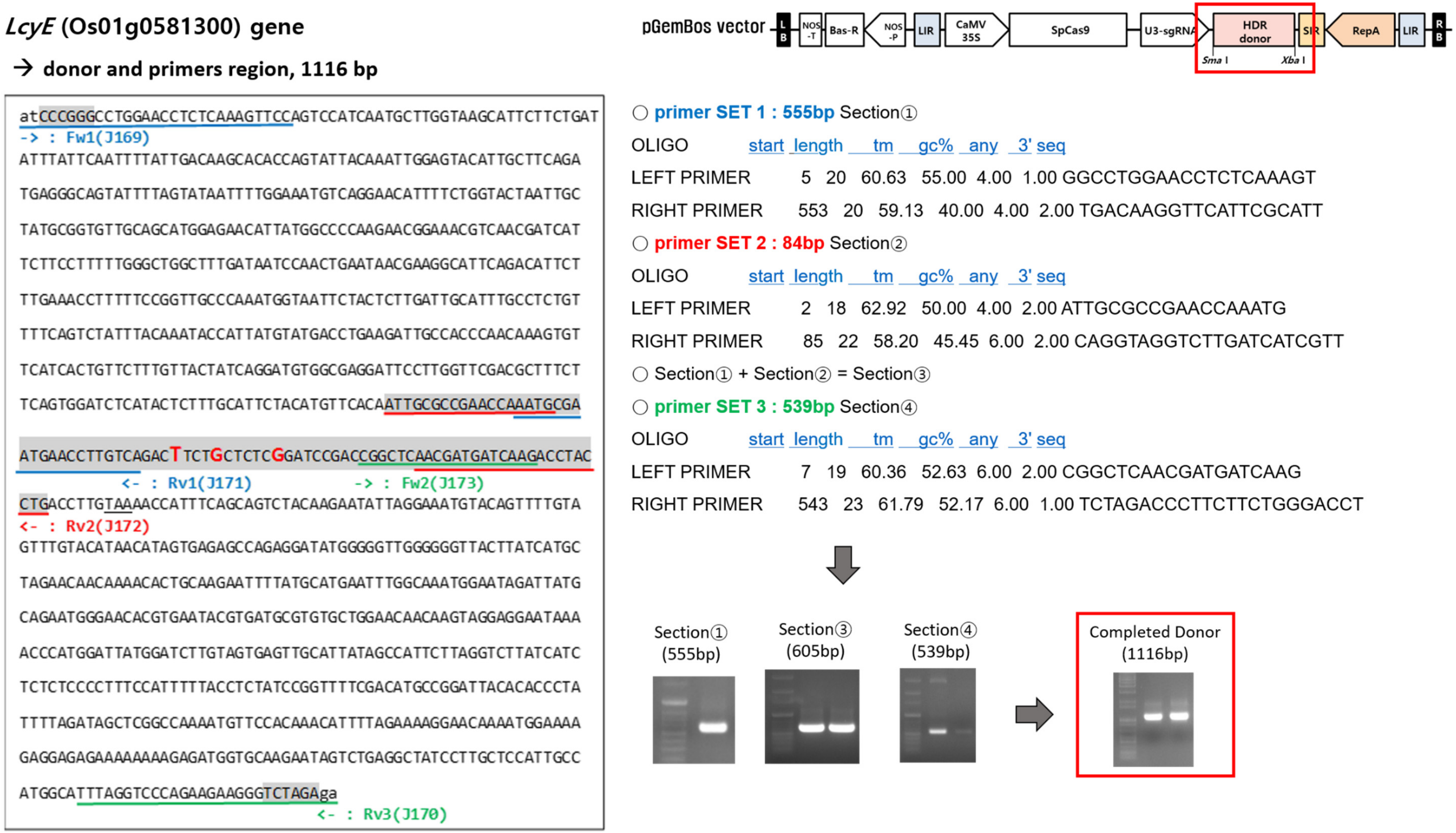

Supplementary Figure S1. Design of donor template using the CRSPR-Cas9 and geminiviral replicon system.

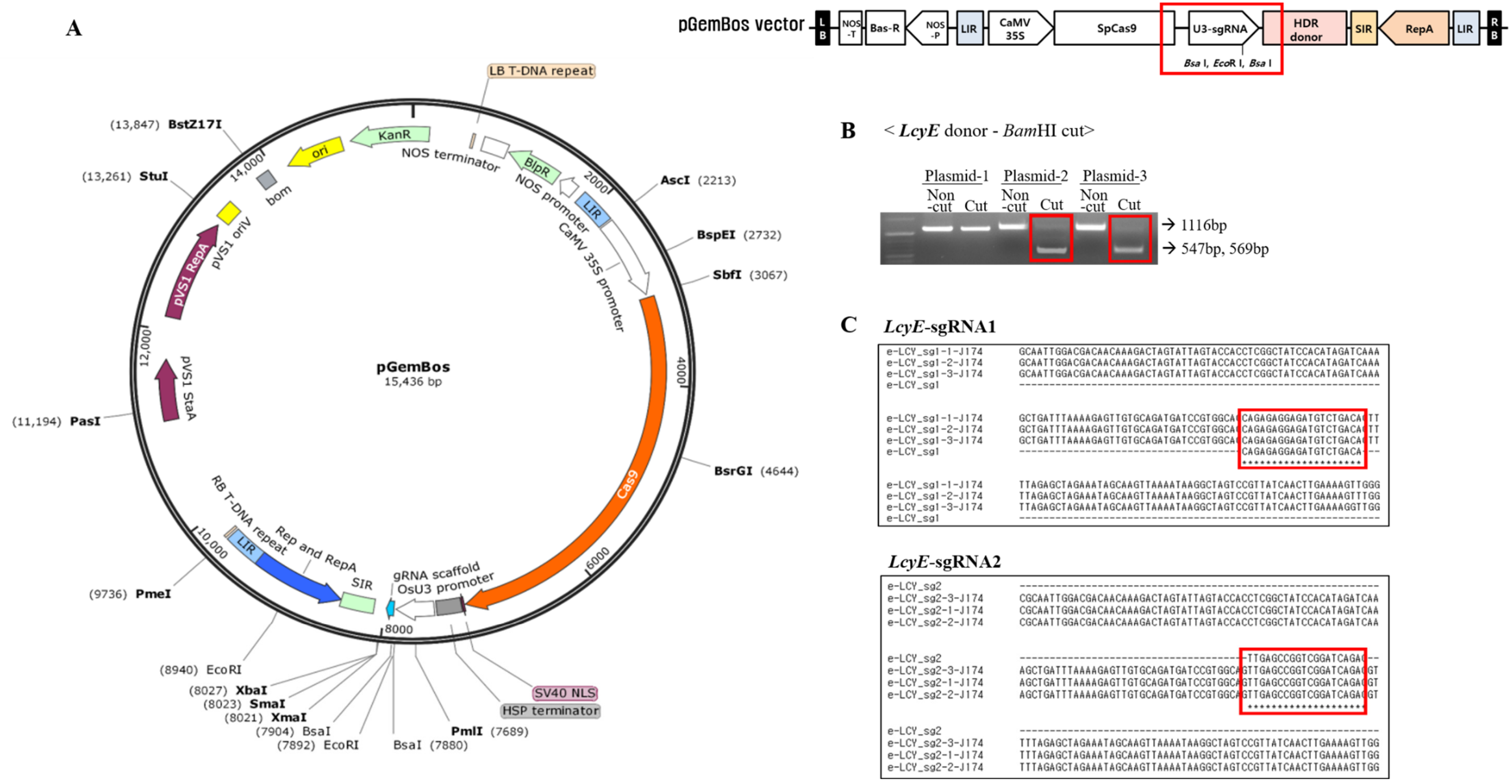

Supplementary Figure S2. Ti-plasmid vector construction using the CRSPR-Cas9 and geminiviral replicon system (A) pGemBos vector construction (B) Confirmation of donor template into pGemBos::*LcyE* vector (C) Confirmation of sgRNA by sequencing analysis.

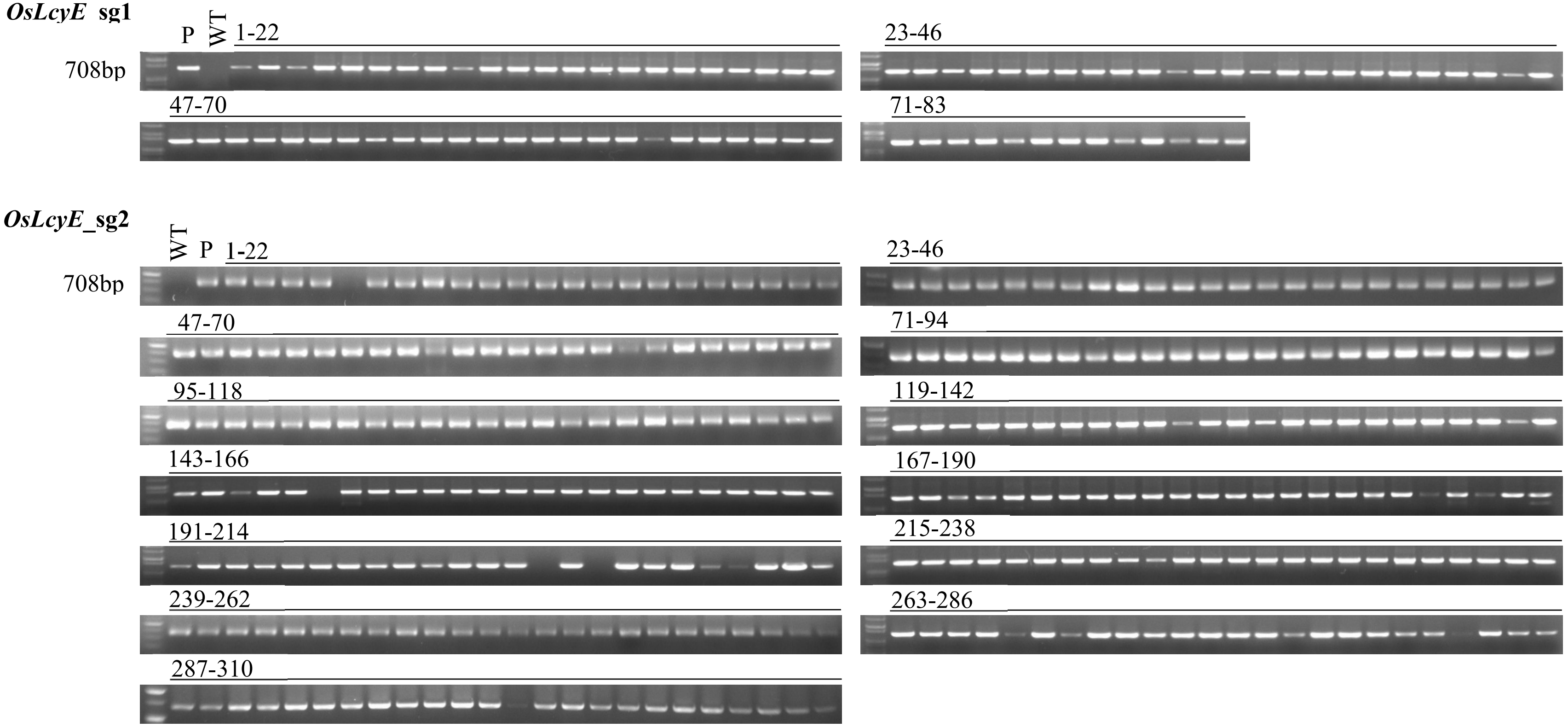

Supplementary Figure S3. Confirmation of T-DNA insertion from calli obtained through HDR experiment. M: 1kb DNA ladder; WT: wild-type; P: pGemBos::*LcyE* plasmid vector.

Supplementary Table S2. The primers list used in this study.

Primer name	Sequence (primer direction 5'-3')
LcyE donor 1st Fw	TCCTGCAACCGGTACTAACA
LcyE donor 1st Rv	GTGGTGCAAGGAAGGAGAAG
LcyE donor 2nd Fw	ATCCCGGGCCTGGAACCTCTCAAAGTTCC
LcyE donor 2nd Rv	TCTCTAGACCCTTCTTCTGGGACCTAAA
LcyE donor OE-PCR Fw1	CGGCTCAACGATGATCAAG
LcyE donor OE-PCR Rv1	TGACAAGGTTCATTCGCATT
LcyE donor OE-PCR Rv2	CAGGTAGGTCTTGATCATCGTT
T-DNA confirm-Nos ter Fw	TTGCGCGCTATATTTTGTTTT
T-DNA confirm-Bar R Rv	CGTCAACCACTACATCGAGA
Replicon confirm-Rep Fw	TTCTCCCAGAGAAACTGGAA
Replicon confirm-35S Rv	CCATCTGTGGGTTAGCATTC
NGS (Next Generation Sequencing) primers	
LcyE 1st Fw	TTCAATTCTTGCAAATTTCTTCA
LcyE 1st Rv	TCATTTTTGGTCTAGTTACACATCA
LcyE 2nd Fw	ACACTCTTTCCCTACACGACGCTCTTCCGATCTTCTTTTTGGCGTATGAGCAG
LcyE 2nd Rv	GTGACTGGAGTTCAGACGTGTGCTCTTCCGATCTGGTCATGTCCTCGGTGAACT
LcyE 3rd-1	AATGATACGGCGACCACCGAGATCTACACtatagcctACACTCTTTCCCTACACGAC
LcyE 3rd-2	AATGATACGGCGACCACCGAGATCTACACatagaggcACACTCTTTCCCTACACGAC

LcyE 3rd-3	AATGATACGGCGACCACCGAGATCTACACcctatcctACACTCTTTCCCTACACGAC
LcyE 3rd-4	AATGATACGGCGACCACCGAGATCTACACggctctgaACACTCTTTCCCTACACGAC
LcyE 3rd-5	AATGATACGGCGACCACCGAGATCTACACaggcgaagACACTCTTTCCCTACACGAC
LcyE 3rd-6	AATGATACGGCGACCACCGAGATCTACACtaatcttaACACTCTTTCCCTACACGAC
LcyE 3rd-7	AATGATACGGCGACCACCGAGATCTACACcaggacgtACACTCTTTCCCTACACGAC
LcyE 3rd-8	AATGATACGGCGACCACCGAGATCTACACgtactgacACACTCTTTCCCTACACGAC
LcyE 3rd-9	CAAGCAGAAGACGGCATACGAGATcgagtaatGTGACTGGAGTTCAGACGTGT
LcyE 3rd-10	CAAGCAGAAGACGGCATACGAGATtctccggaGTGACTGGAGTTCAGACGTGT
LcyE 3rd-11	CAAGCAGAAGACGGCATACGAGATaatgagcgGTGACTGGAGTTCAGACGTGT
LcyE 3rd-12	CAAGCAGAAGACGGCATACGAGATggaatctcGTGACTGGAGTTCAGACGTGT
LcyE 3rd-13	CAAGCAGAAGACGGCATACGAGATttctgaatGTGACTGGAGTTCAGACGTGT
LcyE 3rd-14	CAAGCAGAAGACGGCATACGAGATacgaattcGTGACTGGAGTTCAGACGTGT
LcyE 3rd-15	CAAGCAGAAGACGGCATACGAGATagcttcagGTGACTGGAGTTCAGACGTGT
LcyE 3rd-16	CAAGCAGAAGACGGCATACGAGATgcgcattaGTGACTGGAGTTCAGACGTGT
LcyE 3rd-17	CAAGCAGAAGACGGCATACGAGATcatagccgGTGACTGGAGTTCAGACGTGT
LcyE 3rd-18	CAAGCAGAAGACGGCATACGAGATttcgcggaGTGACTGGAGTTCAGACGTGT
LcyE 3rd-19	CAAGCAGAAGACGGCATACGAGATgcgcgagaGTGACTGGAGTTCAGACGTGT
LcyE 3rd-20	CAAGCAGAAGACGGCATACGAGATctatcgctGTGACTGGAGTTCAGACGTGT
